# Fibroblast-centered mechanisms of chronic inflammation and airway remodeling in allergic bronchopulmonary aspergillosis

**DOI:** 10.3389/falgy.2026.1952768

**Published:** 2026-09-11

**Authors:** Menglin Li, Chenxiao Qiao, Yun Pan, Jintao Zhang, Qian Chen, Ying Wang, Yinghui Qu, Ruichen Du, Shizhen Wang, Qian Qi

**Affiliations:** 1 Department of Respiratory, The First Affiliated Hospital of Shandong First Medical University & Shandong Provincial Qianfoshan Hospital, Shandong Institute of Respiratory Diseases, Featured Laboratory of Respiratory Immunology and Regenerative Medicine in Universities of Shandong, Jinan Clinical Research Center for Respiratory Disease, Jinan, Shandong, China; 2 Shandong Provincial Key Medical and Health Laboratory of Translational Medicine in Microvascular Aging, The First Affiliated Hospital of Shandong First Medical University & Shandong Provincial Qianfoshan Hospital, Jinan, Shandong, China

**Keywords:** airway remodeling, allergic bronchopulmonary aspergillosis, bronchiectasis, fibroblast, myofibroblast, type 2 inflammation

## Abstract

Allergic bronchopulmonary aspergillosis (ABPA) is a complex hypersensitivity lung disease driven predominantly by type 2 immune responses. However, the classical immunological paradigm fails to adequately explain the persistent airway structural damage and frequent relapses observed even after inflammatory control is achieved. This review proposes a fibroblast-centered mechanistic framework that synthesizes evidence from ABPA, fungal-associated airway diseases, and pulmonary fibrosis to systematically delineate the mechanisms linking fibroblasts to chronic inflammation and airway remodeling. In the context of chronic inflammation and persistent epithelial injury, fibroblasts can be activated by various signals, including type 2 cytokines such as interleukin-4 (IL-4) and IL-13. Upon activation, fibroblasts exert their effects through functionally distinct subpopulations: they actively recruit eosinophils through chemokine secretion (e.g., CCL11), establishing a positive feedback loop of “epithelial injury–fibroblast activation–eosinophil infiltration–tissue damage” that sustains and amplifies inflammation. Prolonged activation further drives fibroblast-to-myofibroblast transition, characterized by expression of *α*-smooth muscle actin (*α*-SMA) and deposition of excessive extracellular matrix, resulting in irreversible airway remodeling and central bronchiectasis. Clinically, fibroblast-related markers such as periostin, transforming growth factor-*β*, and *α*-SMA show promise for future risk stratification. Biologics targeting fibrotic pathways have already demonstrated anti-remodeling potential. In the future, the integration of single-cell transcriptomics, spatial omics, and longitudinal clinical studies may clarify the roles of distinct fibroblast subpopulations in ABPA progression and treatment response. This integration could also enable the establishment of a comprehensive “inflammation–remodeling” assessment system—facilitating a therapeutic transition from pure immunosuppression toward combined modulation of tissue repair.

## 1 Introduction

Allergic bronchopulmonary aspergillosis (ABPA) is a complex hypersensitivity lung disease triggered by persistent airway exposure to *Aspergillus* antigens, which most commonly affects patients with asthma or cystic fibrosis. Patients with ABPA show marked clinical heterogeneity. Patients present with chronic cough, wheezing, dyspnea secondary to poorly controlled asthma, and expectoration of brown-colored mucus plugs. Radiologically, fleeting pulmonary infiltrates, bronchiectasis, and mucoid impaction are frequently observed (1). In a subset of patients, sustained airway injury leads to the development of irreversible fibrosis (2), which may ultimately progress to ABPA with chronic pleuropulmonary fibrosis (ABPA-CPF) (3). ABPA is immunologically characterized by predominant type 2 immune responses, featuring enhanced interleukin-4 (IL-4), IL-5, and IL-13 signaling, elevated serum immunoglobulin E (IgE) levels, and eosinophilic inflammation. Although the majority of patients achieve clinical and serological improvement following oral corticosteroid and/or azole antifungal therapy, approximately 40%–50% of patients experience relapse during corticosteroid tapering or after treatment cessation (4–6). The classical type 2 inflammation model cannot sufficiently account for persistent airway inflammation, recurrent relapse, and irreversible structural damage observed in certain patients after inflammatory control is achieved.

The immune–stromal cell crosstalk described in asthma provides an important conceptual framework for understanding the connection between persistent inflammation and structural remodeling in ABPA. In severe asthma, accumulating evidence indicates that bronchial fibroblasts participate in airway remodeling and may also contribute to maintaining chronic airway inflammation through aberrant proliferation, enhanced migration or invasive capacity, and excessive extracellular matrix (ECM) production. Studies on lung injury and pulmonary fibrosis have shown that fibroblasts produce various inflammatory mediators, including CCL11. CCL11 not only modulates fibroblast proliferation and collagen synthesis (7) but also participates in the recruitment, activation, and tissue retention of immune cells, such as eosinophils (8, 9), thus amplifying type 2 immune responses.

Severe asthma and ABPA share multiple overlapping immunopathological features, including type 2 immune responses, eosinophilia, mucus hypersecretion, and airway remodeling (10, 11). These commonalities suggest that the aberrant fibroblast phenotypes documented in severe asthma may also be present in ABPA, although direct evidence remains to be established. Compared with severe asthma, ABPA is characterized by persistent or recurrent exposure to *Aspergillus* antigens within the airways; some patients exhibit airway colonization or sustained fungal growth within mucus plugs (12, 13). This creates a state of continuous antigenic stimulation and repetitive injuries to the epithelium. Such persistent insults are likely to drive sustained—and potentially dysregulated—fibroblast activation, thereby amplifying both the immune responses and structural damage. In this context, fibroblasts may emerge as active drivers of a self-perpetuating inflammatory cycle that links fungal persistence, airway injury, immune activation, and airway remodeling.

Although the involvement of fibroblasts in asthma-related airway remodeling and chronic inflammation has received considerable attention, the activation status, functional subsets, immunomodulatory roles, and relationship with bronchiectasis of fibroblasts in ABPA remain poorly characterized. We hypothesize that recurrent fungal exposure and epithelial injury in ABPA may induce distinct fibroblast subpopulations with chemokine-producing, ECM-producing, or myofibroblastic features, which collectively sustain inflammation and structural remodeling. This review integrates existing evidence from ABPA, fungal-associated airway diseases, severe asthma, and pulmonary fibrosis, focusing on the following: how persistent *Aspergillus* exposure and epithelial injury trigger fibroblast activation; how fibroblasts interact with type 2 immune responses and eosinophils; and how these processes promote chronic inflammation, ECM remodeling, and bronchiectasis. Given the current lack of direct ABPA-specific data, we further propose a fibroblast-centered mechanistic framework that is suitable for future experimental and clinical validation.

## 2 The classical immune paradigm of ABPA and its limitations

The hallmark of ABPA is an aberrant antifungal immune response rather than an invasive fungal infection, typically initiated by abnormal immune recognition of *Aspergillus* antigens in genetically susceptible individuals (11). Persistent airway colonization by *Aspergillus* or repeated antigen exposure is considered a critical driver of chronic immune activation in ABPA (14). Fungal proteases persistently disrupt the airway epithelial barrier, inhibit phagocyte activity, and impair mucociliary clearance. Concurrently, defective mucociliary function and an abnormal mucus milieu may further promote *Aspergillus* antigen persistence, establishing a chronically immunostimulatory environment (15, 16). Disruption of the epithelial barrier plays a critical role in the initiation and exacerbation of allergic diseases (17). Accordingly, the airway epithelium is repeatedly damaged by *Aspergillus fumigatus*, leading to the release of alarmins such as IL-25, IL-33, and thymic stromal lymphopoietin (TSLP) (18). These alarmins activate group 2 innate lymphoid cells (ILC2s) to rapidly produce type 2 cytokines, driving early acute inflammation (19, 20), while also priming dendritic cells to induce CD4⁺ T-cell differentiation toward the Th2 phenotype (21, 22). Subsequently, Th2 cells produce large quantities of IL-4, IL-5, and IL-13, which are key type 2 cytokines that mediate IgE production, eosinophil recruitment, and mucus hypersecretion (23). Specifically, IL-4 and IL-13 drive B cells to generate *Aspergillus*-specific and total IgE (24); IL-13 additionally promotes mucus hypersecretion, aberrant epithelial responses, and fibroblast activation, thereby contributing to chronic airway remodeling. Enhanced IL-4 signaling facilitates B-cell immunoglobulin class switching to IgE (23). Eosinophils serve as one of the principal effector cells in ABPA. Driven by IL-5 and eotaxin signals, they are massively recruited and accumulate within the airway lumen, forming mucus plugs and releasing cytotoxic granule proteins such as major basic protein and eosinophil peroxidase, which further exacerbate epithelial injury and inflammatory amplification (25).

However, the classical type 2 inflammation-centric model of ABPA has notable limitations. It inadequately addresses central bronchiectasis, mucus impaction, and irreversible airway remodeling, which are characteristic of ABPA. These features may not solely arise from excessive type 2 immune responses but may also involve sustained stromal activation, driving a vicious cycle of persistent injury and aberrant repair. Moreover, this model cannot fully explain why some patients relapse despite improved inflammatory markers or why patients with comparable inflammatory burdens exhibit divergent structural damage. These observations suggest the involvement of non-type 2 pathways in driving inflammation and distinct fibroblast subpopulations in patients. Collectively, these unresolved issues indicate that non-immune structural cells, particularly fibroblasts, may serve as additional regulatory nodes linking inflammation and airway remodeling.

## 3 *Aspergillus*-induced epithelial injury as an initiating signal for fibroblast activation

### 3.1 Distinct morphological forms of *A. fumigatus* in epithelial barrier disruption

*A. fumigatus* conidia represent the primary form through which the host encounters the fungus, initially interacting with airway epithelial cells (AECs) before germinating into hyphae and releasing the fungal products. The airway epithelium serves not only as a physical barrier but also as a critical sensory interface for fungal recognition and initiation of immune responses.

Conidia and hyphae of *A. fumigatus* disrupt the epithelial barrier through distinct mechanisms: conidia activate epithelial damage-associated signaling pathways via contact-dependent mechanisms, inducing the secretion of pro-inflammatory cytokines, such as IL-8 and granulocyte-macrophage colony-stimulating factor (GM-CSF), initiating epithelial injury, and disrupting intercellular adhesion (26). *In vitro* and *in vivo* models have shown that conidial exposure alters expression of epithelial pattern recognition receptor (PRR) (e.g., Dectin-1) and contributes to epithelial injury. Germination and hyphal elongation perpetuate cellular injury signals, exacerbate epithelial injury (27), and physically penetrate the epithelial layer (26). Through the concerted action of multiple morphological forms and mechanisms, *A. fumigatus* ultimately achieves a complete breakdown of the epithelial barrier. Additional studies have demonstrated that fungus–epithelium interactions can alter cell death and stress response patterns, favoring fungal persistence and modulating the local inflammatory milieu (27, 28), further contributing to the disruption of the epithelium.

### 3.2 *Aspergillus*-derived proteases disrupt epithelial integrity and provide inflammatory signaling

Proteases derived from *A. fumigatus* are recognized as critical virulence factors that link fungal exposure to host inflammatory responses. In both animal models and human studies, protease activity has been associated with airway hyperreactivity, epithelial injury, and severe airway inflammation (29). Intranasal administration of *A. fumigatus* culture filtrates in mice has been shown to elicit type 2 immune responses, with fungal proteases playing a predominant role in airway remodeling (30).

Proteases and gliotoxins secreted by mature *A. fumigatus* hyphae act synergistically. They directly lyse epithelial cells by activating injury-related signaling pathways, such as the JNK pathway, and degrade pro-inflammatory factors, including IL-8, thereby disrupting epithelial tissue integrity (26). Concurrently, the secreted metalloprotease Asp f 5 (Mep) exhibits collagenolytic and elastinolytic activity, suggesting that *Aspergillus* may not only induce inflammation but also directly disrupt airway ECM homeostasis, setting the stage for subsequent structural remodeling (31). *Aspergillus* species produce a range of other proteases, the specific host effects of which remain to be fully characterized (32). Certain fungal proteases can also impair host PRR function; for instance, by compromising Dectin-1-dependent antifungal recognition, thereby altering host immune responses to the fungus (33). Furthermore, *Aspergillus*-derived proteases drive mucus production, and mucus hypersecretion may support the growth of inhaled conidia in asthmatic lungs while allowing sustained secretion of proteolytically active allergens, thereby perpetuating a cycle of injury and repair that leads to loss of epithelial integrity and long-term airway architectural alterations (34). Collectively, *Aspergillus* proteases function not only as allergens in immune activation but also as disruptors of the epithelial barrier and modifiers of the tissue microenvironment, providing critical signals for subsequent stromal cell activation and airway remodeling.

Thus, *A. fumigatus* not only damages the airway epithelium in patients with ABPA by inducing excessive type 2 immune responses but also directly compromises epithelial barrier integrity through diverse morphological and mechanistic routes.

### 3.3 *Aspergillus*-driven epithelial–stromal interactions promote airway remodeling

The early interaction between *A. fumigatus* and the airway epithelium determines not only the initial immune activation but may also initiate subsequent persistent inflammation and aberrant repair processes. Epithelial injury is a critical link between fungal exposure and stromal responses. The airway epithelium communicates reciprocally with the underlying mesenchymal cells through what has been termed the “epithelial–mesenchymal trophic unit” (EMTU), reflecting the shaping influence of the epithelium on airway architecture (35). Meanwhile, it is known that in the EMTU of asthma, pro-inflammatory cytokines released by the epithelium can modulate mesenchymal inflammation, resulting in eosinophilia, type 2 inflammation, and neutrophilic inflammation (36). Based on these findings, it is reasonable to speculate on the role of this signaling pathway in the pathogenesis of ABPA.

In most healthy individuals, inhaled conidia are cleared by the innate immune system through phagocytosis. In immunocompromised patients or those with impaired pulmonary function, however, conidial clearance is less effective, permitting germination, prolonged host–allergen exposure, fungal colonization, and epithelial injury (37). Injured epithelial cells release various alarmins and pro-fibrotic mediators, including IL-33, IL-25, TSLP, and other cytokines (18, 38). These signals not only promote type 2 immune responses—leading to airway remodeling and inflammatory alterations (38, 39)—but also act directly on fibroblasts, promoting their activation, migration, and ECM production. Specifically, IL-25 can directly promote fibroblast proliferation, activation, and differentiation (40) and also act through ILC2-dependent indirect pathways (41). IL-33 can not only predominantly exert pro-fibrotic effects indirectly through M2 macrophages and ILC2s but can also act directly on fibroblasts (40). TSLP directly stimulates fibroblasts and promotes CCL2 expression, establishing a chemokine microenvironment that drives sustained fibroblast activation and scar formation (41). These observations underscore that the airway epithelium is not only a structural barrier but also plays a pivotal role in ABPA pathogenesis, including fibroblast-mediated structural remodeling. Subsequent immune cell recruitment and plasma protein extravasation promote a vicious cycle of inflammation, fibrin deposition, and structural alterations. Although direct ABPA-specific evidence remains limited, proteolytically active *A. fumigatus* allergens and metabolic byproducts likely play important roles in disrupting epithelial integrity, driving mucin production, subepithelial fibrosis, and smooth muscle hyperreactivity (29, 34).

In summary, the role of *A. fumigatus* in ABPA likely extends beyond serving as a source of antigen stimulation. *Aspergillus*-mediated epithelial injury, barrier disruption, and aberrant repair may create a microenvironment that continuously activates mesenchymal cells. In this process, the airway epithelium may function as a critical link between fungal exposure and fibroblast activation, laying the foundation for subsequent chronic inflammatory cycles and irreversible airway remodeling.

## 4 Lung fibroblasts as immune-regulatory cells: beyond structural support

Fibroblasts have long been considered to be primarily responsible for synthesizing and remodeling the ECM to maintain tissue structural integrity. However, recent single-cell sequencing studies have revealed that fibroblasts are not a homogeneous population of structural cells; instead, they represent a group of stromal cells with high functional heterogeneity that can actively participate in tissue repair (42). Furthermore, ECM synthesized by lung fibroblasts can convey environmental cues to cells, thereby shaping cellular behavior (43). Recent investigations in *Aspergillus* exposure or infection models have shown that lung stromal cells undergo state reprogramming, giving rise to novel activated fibroblast populations endowed with both immunomodulatory capacity and enhanced ECM production. Notably, the targeted ablation of activated fibroblasts expressing the pro-fibrotic marker periostin (POSTN) in an *A. fumigatus* infection model led to impaired host defense, suggesting that specific fibroblast subpopulations not only participate in tissue remodeling but also play important roles in maintaining antifungal immune homeostasis (44). In recent years, single-cell transcriptomic studies have transformed our understanding of the fibroblasts. Pulmonary fibroblasts are not a uniform population; they comprise subpopulations with distinct functional characteristics, such as remodeling fibroblasts involved in ECM deposition, and inflammatory fibroblasts with chemokine-producing capacity (45–48).

Various respiratory injury factors can induce functional reprogramming of fibroblasts, participating in both acute injury repair and chronic disease progression through shared or overlapping inflammatory signaling pathways. In acute settings, the immunological contributions of fibroblasts range from immune cell recruitment and activation to immunosuppression and inflammation. By expressing chemokines and adhesion-related molecules, fibroblasts provide tissue-positioning signals to circulating immune cells and influence the type and functional state of recruited cells, playing important immunological roles in acute inflammation, pulmonary infection, and respiratory distress syndrome. Fibroblasts have been implicated in diverse pathological processes in chronic pulmonary diseases. For instance, in asthma, aberrantly activated fibroblasts promote airway inflammation and remodeling; in chronic obstructive pulmonary disease (COPD), abnormal fibroblast states correlate with structural alterations and disease severity; and in idiopathic pulmonary fibrosis (IPF), distinct fibroblast subpopulations are closely associated with fibrotic progression (49). Although direct studies on fibroblasts from patients with ABPA remain scarce, evidence from fungal infections, severe asthma, and pulmonary fibrosis suggests that persistent fungal antigen stimulation and a type 2 inflammatory milieu in ABPA may drive the functional reprogramming of fibroblasts. This potential mechanism may partially explain the persistent airway injury and progression of fibrosis in patients with severe ABPA. In this process, fibroblasts may concurrently assume both inflammatory regulatory and structural remodeling functions, positioning them as key cellular nodes linking immune dysregulation to irreversible airway injuries.

## 5 Fibroblast-centered mechanisms of chronic inflammation and airway remodeling in ABPA

### 5.1 Multiple signals drive fibroblast activation

In the context of chronic inflammation and persistent epithelial injury, fibroblasts can be activated by various signals originating from multiple sources, leading to functional reprogramming. These signals include paracrine factors released by immune cells, damage-associated molecular patterns (DAMPs), fungal-derived molecules, and alterations in the local tissue microenvironment. These stimuli activate fibroblasts through PRRs and various inflammatory and fibrotic signaling pathways (50, 51). Examples include type 2 cytokines IL-4 and IL-13 secreted by Th2 cells (49, 52); pro-fibrotic factors and growth factors such as IL-13 and transforming growth factor-*β*1 (TGF-*β*1) released by macrophages and other cells (53, 54); alarmins (IL-33, IL-25, and TSLP) released by injured epithelial cells (18, 38); and fungal-derived molecules recognized by PRRs, triggering the release of pro-inflammatory cytokines and chemokines (55). Importantly, fibroblast activation does not represent a single terminal state but rather a dynamic transition between different functional phenotypes. For instance, fibroblasts express multiple PRRs, including Toll-like receptors (TLRs). TLR ligands can activate fibroblasts, promoting their differentiation into myofibroblasts, thereby enhancing collagen production and driving fibrotic progression (50, 54, 55).

### 5.2 Bidirectional crosstalk between fibroblasts and type 2 inflammation

ABPA is immunologically characterized by type 2 immune responses, in which type 2 cytokines, such as IL-4 and IL-13, not only mediate IgE production and eosinophilic inflammation but also directly affect fibroblast functional status (56, 57). Both IL-4 and IL-13 promote fibroblast invasion and proliferation (49, 52). IL-13 is also a potent stimulator of fibroblast proliferation and collagen production. It can enhance TGF-*β* production by macrophages and epithelial cells, influence TGF-*β* activation, and directly affect myofibroblast differentiation (57, 58). TGF-*β* is a key activator of fibroblasts, promoting their proliferation and stimulating ECM synthesis (59). In addition, IL-13 promotes pulmonary fibroblast-to-myofibroblast transition, as evidenced by increased *α*-smooth muscle actin (*α*-SMA) and collagen expression (60), contributing to airway remodeling.

Contrary to the traditional view that fibroblasts are merely recipients of type 2 inflammatory signals, they can actively regulate the recruitment and localization of immune cells in tissue. Through chemokine production, activated fibroblasts promote eosinophil migration into airway tissues and sustain the local inflammation. Substantial evidence documents fibroblasts as a cellular source of CCL11 (also known as eotaxin-1); *in vitro* treatment of fibroblasts with bleomycin induces CCL11 production (8), which exhibits potent and selective eosinophil chemotactic activity (9). In the context of allergic responses, activated eosinophils induce tissue damage (61). Eosinophils are not merely terminal effector cells recruited by Th2 responses in allergic reactions but can rapidly secrete pre-formed Th2-initiating cytokines, including IL-4 and IL-13, upon stimulation, further reinforcing Th2 immunity (62).

Collectively, key cytokines of type 2 immune responses activate fibroblasts, which in turn recruit eosinophils to further amplify type 2 immune responses. This bidirectional interaction propagates and amplifies the effects of type 2 inflammation.

### 5.3 Fibroblast-driven inflammatory amplification loop in ABPA

In ABPA, *A. fumigatus* antigens persist in an abnormal mucus environment, inducing sustained immune activation in susceptible hosts (63). Barrier damage caused by fungal proteases and fungus–epithelium interactions further promotes antigen exposure and inflammatory maintenance (27). These persistent antigenic stimuli and repetitive epithelial injuries activate fibroblasts. Subsequently, activated fibroblasts actively participate in immune regulation by secreting chemokines, including CCL11, which promote the recruitment and retention of inflammatory cells, particularly eosinophils (8, 9).

Upon stimulation by *A. fumigatus*, eosinophils release extracellular DNA traps [eosinophil extracellular traps (EETs)]. EETs are capable of trapping *A. fumigatus* conidia but do not exert fungicidal effects against them. Moreover, excessive release of EETs may contribute to mucus formation, leading to airway obstruction and impaired pulmonary function. Furthermore, free extracellular granules (FEGs) are anchored to EETs (64). Eosinophils contain approximately 200 granules that serve as pre-formed storage depots for specific granule proteins (14). Highly cationic granule proteins exert cytotoxicity through multiple mechanisms: disrupting lipid bilayer integrity, exerting neurotoxic properties, manifesting RNase activity, and participating in the generation of reactive oxygen species and free radicals, thereby perpetuating inflammation and tissue destruction (65, 66). Consequently, ABPA may involve a positive feedback loop sustained by fibroblasts: persistent *Aspergillus* exposure and epithelial injury induce fibroblast activation; activated fibroblasts release chemokines, such as CCL11, promoting eosinophil recruitment; eosinophils release cytotoxic granule proteins, causing further tissue injury; and injury leads to enhanced fungal antigen exposure and sustained fibroblast activation.

### 5.4 Fibroblast-to-myofibroblast transition in airway remodeling and bronchiectasis

Central bronchiectasis in ABPA likely represents the superposition of two distinct processes: reversible dilation due to mucus plugging and airway obstruction, and irreversible dilation resulting from chronic inflammation, ECM remodeling, and destruction of airway wall architecture. The irreversible component likely arises from persistent inflammation and repeated epithelial injury, which activates fibroblasts and exacerbates type 2 immune responses. Activated fibroblasts can undergo fibroblast-to-myofibroblast transition. Myofibroblasts exhibit excessive ECM deposition, leading to permanent airway remodeling. Thus, bronchiectasis in ABPA may be the net result of reversible obstructive changes combined with irreversible, fibroblast-driven structural alterations.

Initially, *A. fumigatus* infection in patients with ABPA interacts with epithelial cells, causing sustained inflammation and epithelial injury (27). Fibroblasts are then activated by abundant pro-fibrotic factors and growth factors—notably TGF-*β*1, platelet-derived growth factor (PDGF), and IL-13—which serve as key activating signals (53, 54). Activated fibroblasts transition into myofibroblasts during migration along fibrin grids, and TGF-*β* released by inflammatory cells and injured epithelial cells strongly induces the fibroblast-to-myofibroblast transition (67). This transformation proceeds through two stages: fibroblasts first develop an intermediate phenotype termed proto-myofibroblasts, which then differentiate into mature myofibroblasts. In addition, myofibroblasts may also be derived from the recruitment and transformation of circulating fibrocytes (47). Ultimately, transformed myofibroblasts acquire contractile and secretory functions, begin to proliferate, express *α*-SMA (67), and possess the capacity to synthesize and secrete substantial ECM components, including type I collagen, type III collagen, and fibronectin (FN1), leading to the accumulation of collagen fibers around the bronchi of all sizes (68). Myofibroblasts are also sources of matrix metalloproteinases (MMPs) and their inhibitors [tissue inhibitors of metalloproteinases (TIMPs)]. In asthma, myofibroblasts secrete both MMPs and TIMPs, but the MMP/TIMP ratio is reduced (with a relative increase in the TIMP levels) (69), accompanied by collagen deposition, subepithelial fibrosis, airway wall thickening, and stiffness, culminating in airway remodeling. As persistent inflammation drives ongoing remodeling, collagen deposition makes the airway wall rigid and fragile. Structural support is progressively destroyed, and the airway undergoes permanent, irreversible changes due to the combined effects of inflammatory destruction and aberrant repair, ultimately resulting in bronchiectasis.

**Figure 1:**
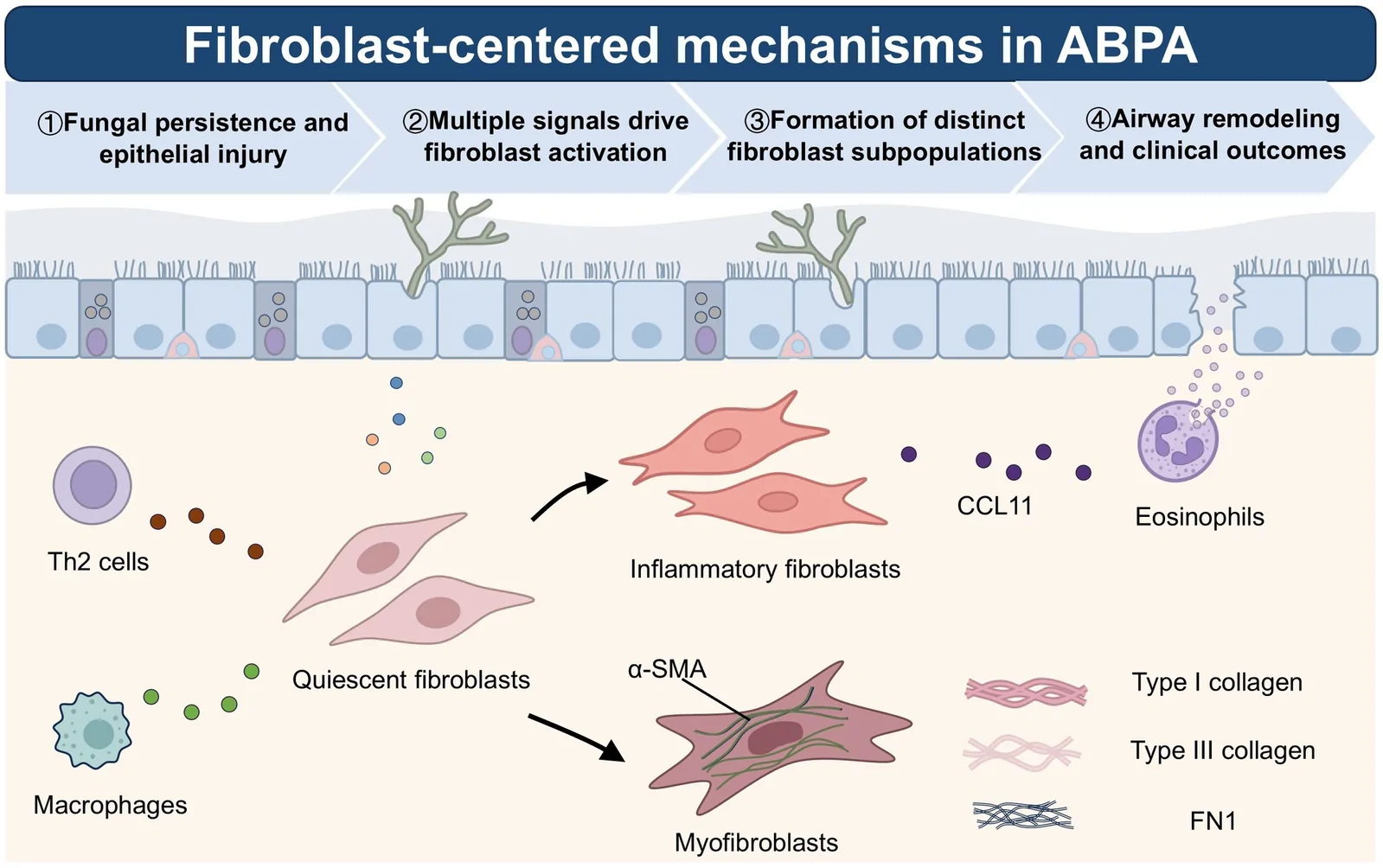
Fibroblast-centered mechanisms in ABPA.

In summary, airway remodeling in ABPA is unlikely to result solely from recurrent mucus obstruction or inflammatory injury but rather represents a dynamic process driven by persistent fungal stimulation, type 2 immune responses, and aberrant fibroblast activation and transition. In this process, fibroblasts simultaneously assume both inflammatory regulatory and structural remodeling functions, positioning them as a key cellular hub connecting immune dysregulation to irreversible bronchiectasis. Fibroblast-centered mechanisms in ABPA are presented in Table 1 and Figure 1.

**Table 1:** Fibroblast-centered mechanisms in ABPA.

Stage	Core pathological events
(1) Fungal persistence and epithelial injury	a. Exposure to *A. fumigatus* conidia contributes to epithelial injury. b. Germination of *A. fumigatus* conidia and subsequent hyphal extension aggravate epithelial injury, with hyphae physically penetrating the epithelial layer. c. Proteases and gliotoxins secreted by mature *A. fumigatus* hyphae synergistically disrupt epithelial integrity.
(2) Multiple signals drive fibroblast activation	a. Injured epithelial cells release abundant alarmins, including IL-33, IL-25, and TSLP. b. Macrophages and other cell types secrete pro-fibrotic mediators and growth factors, including IL-13 and TGF-*β*1. c. Th2 cells secrete type 2 cytokines, including IL-4 and IL-13. d. Fungal-derived molecules also participate in this process.
(3) Formation of distinct fibroblast subpopulations	a. Inflammatory fibroblasts actively recruit eosinophils by secreting chemokines, such as CCL11, leading to epithelial injury and amplification of inflammation. b. Myofibroblasts express *α*-SMA and extensively synthesize and secrete ECM components, such as type I collagen, type III collagen, and FN1, leading to excessive collagen deposition and airway remodeling.
(4) Airway remodeling and clinical outcomes	a. Patients present with chronic cough, wheezing, dyspnea secondary to poorly controlled asthma, and expectoration of brown-colored mucus plugs. b. Radiologically, fleeting pulmonary infiltrates, bronchiectasis, and mucoid impaction are frequently observed.

## 6 Clinical implications of fibroblast-centered mechanisms in ABPA

### 6.1 Fibroblast-related biomarkers in ABPA

ABPA currently lacks biomarkers capable of simultaneously reflecting both immunoinflammatory activity and the risk of airway structural damage. Given that fibroblasts participate in both inflammatory regulation and ECM remodeling, the identification of fibroblast-related molecules may offer novel directions for risk stratification and long-term management of patients with ABPA. POSTN is an ECM protein that has been extensively studied in various type 2 airway diseases (45, 70). It is induced in bronchial epithelial cells by IL-4 and IL-13 (71) and in mesenchymal cells (including fibroblasts) by TGF-*β* (46). TGF-*β* can interact with POSTN, thereby promoting pulmonary fibrosis (72). POSTN promotes eosinophil tissue infiltration in Th2-associated mucosal inflammation (73) and also participates in regulating collagen deposition, tissue biomechanical properties, and aberrant repair processes (74, 75). Thus, in ABPA, POSTN may serve as a candidate surrogate marker for fibroblast activation and remodeling propensity. However, direct evidence regarding the relationship between POSTN and radiographic progression, bronchiectasis severity, and relapse risk in ABPA remains limited and requires further investigation.

TGF-*β* is a key regulator of fibroblast activation and fibrosis. Under pathological conditions, overexpressed TGF-*β* can induce epithelial–mesenchymal transition (EMT), ECM deposition, and cancer-associated fibroblast (CAF) formation, leading to fibrotic diseases and cancer (72). Changes in TGF-*β* levels may reflect local pro-fibrotic signaling(59). From a clinical perspective, TGF-*β*-related pathways may help identify patients at risk of irreversible structural changes, such as bronchiectasis. Nevertheless, TGF-*β* measurements are affected by various biological factors, and their implementation in routine clinical practice remains challenging.

*α*-SMA is a marker protein that defines the myofibroblast phenotype. This protein is synthesized and released by fibroblasts that have undergone phenotypic transition following induction by inflammatory cytokines and is closely associated with aberrant ECM accumulation and pathological airway tissue restructuring (67). Clinically, *α*-SMA may indirectly reflect aberrant fibroblast activation and pathological airway structural alterations, predicting the potential risk of persistent airway injury and fibrotic progression. However, this marker relies on airway biopsy specimens for quantitative detection, is susceptible to non-specific binding due to protein homology, and can be influenced by short-term inflammatory fluctuations.

Given that ABPA encompasses both immunoinflammatory and structural remodeling features, a single biomarker is unlikely to fully capture the disease status. Future approaches may integrate serum type 2 inflammatory markers [IgE, eosinophils, thymus and activation-regulated chemokine (TARC), etc.], fibroblast-related indices (POSTN, TGF-*β*, and ECM markers), and imaging parameters to establish a comprehensive system for risk stratification.

### 6.2 Fibroblast-targeted therapeutic strategies

Current ABPA treatment primarily targets immune inflammation and fungal burden through corticosteroids, antifungal therapy, and, more recently, developed biologics. However, these strategies are insufficient to completely block long-term structural and airway remodeling. Therefore, modulating fibroblast-related pathways may be a promising future adjunctive therapeutic approach. Key signaling molecules, such as TGF-*β,* play central roles in fibroblast activation and airway remodeling (56). Although no direct therapeutic strategies targeting fibroblast pathways in ABPA are currently available, studies on pulmonary fibrosis, asthma, and other chronic airway diseases suggest that modulating aberrant fibroblast activation may mitigate pathological tissue remodeling (76).

Several drugs have been clinically approved for the treatment of pulmonary fibrosis, and some act directly on fibroblasts. For example, pirfenidone, nintedanib, and nerandomilast are all antifibrotic agents approved by the US Food and Drug Administration (FDA) (77, 78). Pirfenidone is an orally administered synthetic compound. Although its mechanism of action has not been fully elucidated, pirfenidone can inhibit TGF-*β*-induced fibroblast differentiation, proliferation, and collagen production *in vitro* and attenuate both tumor necrosis factor-alpha (TNF-*α*) and IL-1*β* expression and collagen deposition in mouse models of acute lung injury, asthma, and IPF. Studies suggest that this may be related to pirfenidone inhibiting the effects of TGF-*β* by reducing TGF-*β* receptor signaling or by acting further downstream in the signaling pathway (79, 80). Nintedanib is an intracellular tyrosine kinase inhibitor that targets the receptors of vascular endothelial growth factor (VEGF), fibroblast growth factor (FGF), and PDGF. It exerts antifibrotic effects by interfering with fibroblast proliferation, differentiation, and migration, as well as ECM deposition, and also possesses anti-vascular remodeling activity (81). By selectively inhibiting phosphodiesterase 4B (PDE4B), nerandomilast elevates intracellular cyclic adenosine monophosphate (cAMP) levels. On the one hand, it induces the dedifferentiation of pulmonary myofibroblasts; on the other hand, it suppresses vascular leakage by preserving endothelial barrier integrity and inhibits immune cell adhesion to and infiltration into injured endothelial cells (82). These three antifibrotic agents target fibroblast-mediated pulmonary fibrosis from a mechanistic perspective. Nevertheless, it should be noted that the current mainstay therapies for ABPA consist of corticosteroids and antifungal agents, and none of the three agents is routinely recommended by the guidelines for ABPA management. Whether these agents can benefit patients with ABPA and parenchymal pulmonary fibrosis awaits further evidence from clinical trials.

Although current therapeutic approaches for ABPA predominantly target inflammatory components (3), future therapeutic strategies may benefit from approaches that concurrently address fibroblast-mediated tissue remodeling. The biologics currently used in ABPA primarily target IgE or type 2 inflammatory pathways; however, their potential anti-remodeling effects warrant attention. The antifibrotic potential of biologics has been preliminarily validated. For instance, dupilumab [anti-IL-4 receptor *α* subunit (IL-4R*α*)] simultaneously blocks the bioactivity of IL-4 and IL-13 in patients with asthma, significantly reducing type I collagen and fibronectin expression and restoring IL-4-driven fibroblast proliferation to baseline levels (83). Studies have also shown that dupilumab induces rapid and sustained reductions in serum POSTN levels (84), likely reflecting IL-4R*α* blockade-mediated suppression of POSTN expression and ECM generation through the inhibition of IL-4/IL-13 signaling. Mepolizumab (anti-IL-5) treatment in severe asthma reduces submucosal eosinophils, leading to significant improvements in basement membrane thickness and epithelial injury (85). This is likely due to IL-5 pathway blockade reducing eosinophil burden, thereby indirectly attenuating eosinophil-mediated tissue injury and pro-fibrotic stimulation.

Overall, fibroblast-related mechanisms provide a novel theoretical framework for precision therapy of ABPA. Future therapeutic strategies may extend beyond simply suppressing type 2 immune responses toward the dual modulation of immune inflammation and aberrant tissue repair, thereby achieving simultaneous control of disease activity and structural progression.

## 7 Future directions: unraveling fibroblast heterogeneity and therapeutic opportunities in ABPA

Single-cell and spatial transcriptomic technologies, which reveal cellular heterogeneity and spatial tissue architecture at single-cell or spatial resolution, may serve as critical tools for dissecting fibroblast heterogeneity in ABPA. Future studies should determine whether functionally distinct fibroblast subpopulations, such as inflammatory fibroblasts and remodeling fibroblasts, exist in ABPA airway tissues. By integrating molecular features, including POSTN, COL1A1, COL3A1, DCN, CCL11, and CXCL12, different fibroblast states can be further defined. Their spatial relationships with eosinophils, epithelial cells, and immune cells can be analyzed, along with the presence of different fibroblast subpopulations in patients with ABPA with varying inflammatory burdens.

Bronchoalveolar lavage fluid (BALF), obtained via bronchoscopy as a local sample from the lower respiratory tract, directly originates from the alveolar and bronchiolar surfaces and specifically reflects the pulmonary microenvironment. However, as fibroblasts are primarily located in the airway interstitial compartment, BALF does not directly represent fibroblast populations. Nevertheless, the cytokines, chemokines, ECM degradation products, and extracellular vesicles contained may reflect local epithelial–immune–stromal interactions. Future efforts could integrate BALF transcriptomics, proteomics, and serum inflammatory markers to establish a comprehensive scoring system that reflects both “inflammatory status” and “remodeling status.”

ABPA is currently classified based on immunological parameters, radiographic findings, and clinical responses—indices that primarily reflect inflammatory activity but inadequately capture structural remodeling or fibrotic risk. In the future, fibroblast-based endotypes could be developed to address the limitations of existing classifications, which fail to reflect tissue structural damage, thereby providing a complementary perspective for understanding the clinical heterogeneity of ABPA.

Current evidence on fibroblast involvement in ABPA is largely derived from severe asthma, pulmonary fibrosis, and fungal infection models, with direct evidence in patients with ABPA remaining limited. Therefore, longitudinal cohort studies are needed to dynamically evaluate changes in fibroblast-related indices during the disease course and their relationship with IgE levels, eosinophil counts, radiographic progression, and relapse.

Current ABPA treatment primarily targets the fungal burden and type 2 immune responses; however, effective interventions for established structural airway damage are lacking. Future research should explore whether modulating aberrant fibroblast activation, inhibiting excessive ECM deposition, or promoting the reversal of abnormal fibroblast phenotypes could delay bronchiectasis progression and structural remodeling.

## 8 Conclusions and perspectives

Pulmonary fibroblasts are not merely passive cells that maintain tissue architecture; they may also serve as critical regulatory cells linking immune inflammation to airway structural remodeling, playing a pivotal role in sustaining chronic inflammation and driving airway remodeling in ABPA. Although direct functional evidence for fibroblasts in ABPA remains limited, studies on severe asthma, fungal-associated airway diseases, and pulmonary fibrosis collectively suggest that fibroblasts may represent a key cellular hub connecting persistent fungal stimulation, type 2 immune activation and amplification, and irreversible airway injury.

An in-depth elucidation of the functional heterogeneity of fibroblasts and their dynamic interactions with epithelial cells and immune cells in ABPA will facilitate the establishment of novel disease endotypes and advance precision diagnostic and therapeutic strategies. The integration of single-cell transcriptomics, spatial omics, and longitudinal clinical studies holds promise for clarifying the roles of distinct fibroblast phenotypes in ABPA progression and treatment responses.

Accordingly, future ABPA management may need to transition from the pure suppression of type 2 immune responses to integrated strategies that concurrently modulate inflammatory responses and tissue remodeling processes. Interventions targeting fibroblast-related signaling pathways may offer novel therapeutic avenues for the prevention of irreversible airway structural damage.
